# Notes and News

**Published:** 1899-05-13

**Authors:** 


### Notes and News.

(Collected and Communicated.)

### HOSPITAL MEETINGS.

East London Hospital for Children.—Festival Dinner on May 15th, at Whitehall Rooms, Hotel Metropole, Duke of Connaught presiding.

North-Eastern Hospital for Children, Hackney Road, Shoreditch, N.E.—Annual Meeting at half-past four p.m., on Monday, 15th inst., at the Hospital.

Christian Science is by no means extinguished yet by the unpleasant proceedings in which its prophets have taken part. In Chicago several temples have been erected for the furtherance of the tenets.

The owners of buildings arranged as flats are threatened in Paris with a new obligation. This is the compulsory disinfection of every apartment on the removal of a tenant. Whether this regulation will be enforced is doubtful, but it is pretty certain that all dwellings where illness of a notifiable nature has occurred will be subject to compulsory disinfection.

A sanatorium for the treatment of consumption is to be opened at Stoke-by-Nayland, in Suffolk. Mr. Walker, of Dewsbury, the wealthy gentleman who has purchased the site of 94 acres, and will establish the sanatorium, will place it in the charge of his daughter, who is a doctor of medicine. Miss Walker has already conducted a small establishment of the kind at Denver, in Norfolk.

The report of the work of the Italian Red Cross Society in Africa is very interesting. Where other assistance to the wounded is not on a very ample scale the Red Cross Society necessarily plays an important part. A hospital of 50 beds was erected by the Society at Ascari, and worked there for upwards of one year. This hospital established also a kind of out-patients' department. Ten ambulances with attendant doctors were provided to move with the troops. In the various hospital and infirmary stations situated near the seats of war over 2,500 wounded were attended, and many prisoners were also provided with sick accommodation and many necessaries.

The Duke and Duchess of York will open the new building of the Royal London Ophthalmic Hospital, which will provide for 40 additional beds and for a much larger out-patient department. The new building is situated in City Road, about one mile from the present site in Blomfield Street.

The provision of special institution for the reception of consumptive patients under the Poor Law is receiving attention, and at a conference of delegates from Metropolitan Boards of Guardians it was decided to ask the Local Government Board to institute an inquiry into the matter.

The committee in charge of the Lewis Carrol Memorial Fund have endowed an "Alice in Wonderland" Cot at the Glasgow Western Infirmary. The inauguration was performed in the presence of the Duchess of Montrose. Half the amount collected was subscribed by the children attending the day and Sunday schools of Glasgow and the West of Scotland.

The treasurer of St. Thomas's Hospital writes that on May 4th the Right Hon. the Lord Mayor opened a ward here, under the title of "The City of London Ward," to be used for accident cases. The action of the authorities in adopting this name for the ward will, it is hoped, remind the citizens of their heritage in this ancient hospital given them by King Edward VI. so far back as 1553. The ward contains 22 beds, a certain number of which will bear in perpetuity the name of the Mercers' Company as a memorial of the munificence of the senior guild to this hospital in 1898; and it is hoped that before long the remaining beds will bear the names of other guilds or honoured citizens of this great metropolis. The cost of maintenance is estimated at £2,000 per annum, and the authorities trust that the citizens will provide this amount, either by endowment or annual subscriptions, so that the ward may be in reality a "City of London Ward."

A dinner in aid of the Medical Graduates' College and Polyclinic will be held shortly. The college is situated in Chenies Street, Gower Street. The president is Sir William Broadbent, and amongst the vice-presidents are Mr. Jonathan Hutchinson, Professor Burdon Sanderson, and Mr. Thomas Bryant. Mr. William Ord is treasurer and chairman of the council. Dr. Charles Theodore Williams, who is a member of the council, has given £552 for the support of the undertaking, and numbers of other large contributions have been received. The aim of the college is to "facilitate in all directions the lifelong education of medical men, and to offer them opportunities in London which are enjoyed in Vienna, New York, and other capitals." The college will contain a large museum for the display of models, &c., in illustration of disease, and apparatus, instruments, and appliances of the newest patterns. A workroom for clinical and pathological research will be open at all times. It is intended to organise daily consultations for poor persons unable to afford consultants' fees. The consultations would be open to all members of the college. For the special classes and lectures small fees will be charged; and for the maintenance generally of the college the public will be asked to contribute, seeing that they will directly benefit by the improved facilities for knowledge it will afford to medical practitioners.

